# Butyrate as a Potential Driver of a Dysbiotic Shift of the Tongue Microbiota

**DOI:** 10.1128/msphere.00490-22

**Published:** 2022-12-12

**Authors:** Bou-Jon Chen, Toru Takeshita, Teppei Tajikara, Mikari Asakawa, Shinya Kageyama, Yukie Shibata, Yasunori Ayukawa, Yoshitaka Yano, Yoshihisa Yamashita

**Affiliations:** a Section of Preventive and Public Health Dentistry, Division of Oral Health, Growth and Development, Faculty of Dental Science, Kyushu University, Fukuoka, Japan; b Section of Implant and Rehabilitative Dentistry, Division of Oral Rehabilitation, Faculty of Dental Science, Kyushu University, Fukuoka, Japan; c OBT Research Center, Faculty of Dental Science, Kyushu University, Fukuoka, Japan; d Personal Health Care Products Research Laboratories, Kao Corporation, Tokyo, Japan; University of Michigan-Ann Arbor

**Keywords:** butyric acid, tongue microbiota, 16S rRNA

## Abstract

The tongue dorsum is colonized by a stable microbiota, mostly comprising common commensal taxa. However, the predominance of each taxon varies among individuals. We hypothesized that equilibrium in the tongue microbiota is affected by exposure to butyrate in the oral fluid, which is reported to affect the growth of specific microorganisms. In this study, the bacterial composition of the tongue microbiotas of 69 male adults was determined via 16S rRNA gene sequencing to investigate its relationship to *n*-butyric acid concentration in oral rinse samples. The tongue microbiotas of individuals with a higher *n*-butyric acid level had higher relative abundances of Prevotella histicola, Veillonella atypica, and Streptococcus parasanguinis and lower relative abundances of Neisseria subflava and Porphyromonas pasteri. Subsequently, tongue microbiota samples collected from 12 adults were cultivated for 13 h in basal medium containing mucin and different concentrations of sodium butyrate (0, 0.8, 1.6, and 3.2 mM) to assess its effect on the growth of tongue microbiota organisms. The bacterial composition of the cultivated tongue microbiotas also demonstrated a significant gradual shift with an increase in sodium butyrate levels in beta-diversity analysis. *N. subflava* was significantly less predominant in the microbiota after cultivation with an increased addition of sodium butyrate, although no statistical difference was observed in the other aforementioned taxa. These results suggest that butyrate in the oral fluid is partially involved in the dysbiotic shift of the tongue microbiota.

**IMPORTANCE** Oral microbial populations that are always ingested with saliva have attracted increasing attention because more oral microorganisms than previously known reach distal organs, such as the lungs and intestinal tract, thereby affecting our health. However, although such organisms are predominately derived from the tongue dorsum, the dynamics and determinants of the tongue microbiota composition remain unclear. This study demonstrated that exposure to butyrate could lead to a dysbiotic shift in the tongue microbiota using an observational epidemiological and microbiota cultivation approach. This result adds a new dimension to tongue microbiota ecology.

## INTRODUCTION

The human oral cavity is densely colonized by diverse indigenous microorganisms, which are ingested with saliva, beverages, and food. Little attention has been paid to these microbial populations, as they are mostly inactivated by gastric acid and proteolytic enzymes before reaching the gut. However, a recent metagenomic study revealed that more oral commensals than previously assumed reach and colonize the large intestine (1). Other studies have demonstrated that the lungs, which are believed to be sterile, also harbor diverse bacteria that are primarily derived from the oral cavity and can affect respiratory health (2, 3). Thus, the translocation of oral commensals and their association with health have recently attracted increasing attention. Nevertheless, there is still limited information on the compositional dynamics and determinants of the tongue microbiota, which is a primary source of the microbial populations ingested with saliva among distinct microbial communities formed on various oral niches (4–6). Oral microbiota studies have primarily focused on the dental plaque microbiota, which is a causal agent of two major oral diseases, dental caries and periodontitis.

The tongue microbiota is a stable community mostly comprising common commensal taxa. However, the predominance of each taxon in the microbiota varies among individuals. We previously revealed that a shifted equilibrium of these commensals is observed in individuals with poor oral hygiene and more dental caries experience (7, 8). This implies that some compounds enriched in the oral cavity under poor dental conditions may be involved in the composition of the tongue microbiota. Therefore, this study focused on butyrate levels in oral fluids. Butyrate is a metabolite extensively produced by several oral pathobionts, such as Fusobacterium nucleatum and Porphyromonas gingivalis (9), and its concentration in the oral cavity is high in individuals with poor dental conditions (10). Butyrate exhibits antimicrobial effects against several bacterial taxa, including Campylobacter jejuni (11), Helicobacter pylori (12), and oral streptococci such as Streptococcus gordonii and Streptococcus mutans (13). The presence of resistant bacteria, such as P. gingivalis and Aggregatibacter actinomycetemcomitans (13), implies that exposure to butyrate could affect the equilibrium of the tongue microbiota. This observational and epidemiological study investigated the relationship between the bacterial composition of the tongue microbiota and the concentration of *n-*butyric acid in oral rinse samples. Subsequently, we assessed the effects of sodium butyrate on the growth of the tongue microbiota using a microbiota cultivation approach.

## RESULTS

This study investigated the bacterial composition of the tongue microbiota associated with the amount of butyric acid in the oral cavity. Following dental examination, tongue microbiota and oral rinse samples were collected from 69 male adults aged 30 to 59 years. The bacterial composition of the tongue microbiota of these individuals was determined via 16S rRNA gene sequencing using a next-generation sequencer, Ion PGM (Thermo Fisher Scientific), together with that of 60 pre- and postcultivated tongue microbiota samples that were utilized in a subsequent microbiota cultivation analysis. The sequencer generated 1,182,168 quality-filtered reads containing 2,424 distinct sequences (amplicon sequence variants [ASVs]) corresponding to the V1 and V2 regions of the 16S rRNA gene. Of these, 1,753 ASVs containing 1,087,605 reads (92.0%) were assigned to 208 bacterial taxa deposited in eHOMD (14), with ≥98.5% identity.

**FIG 1:**
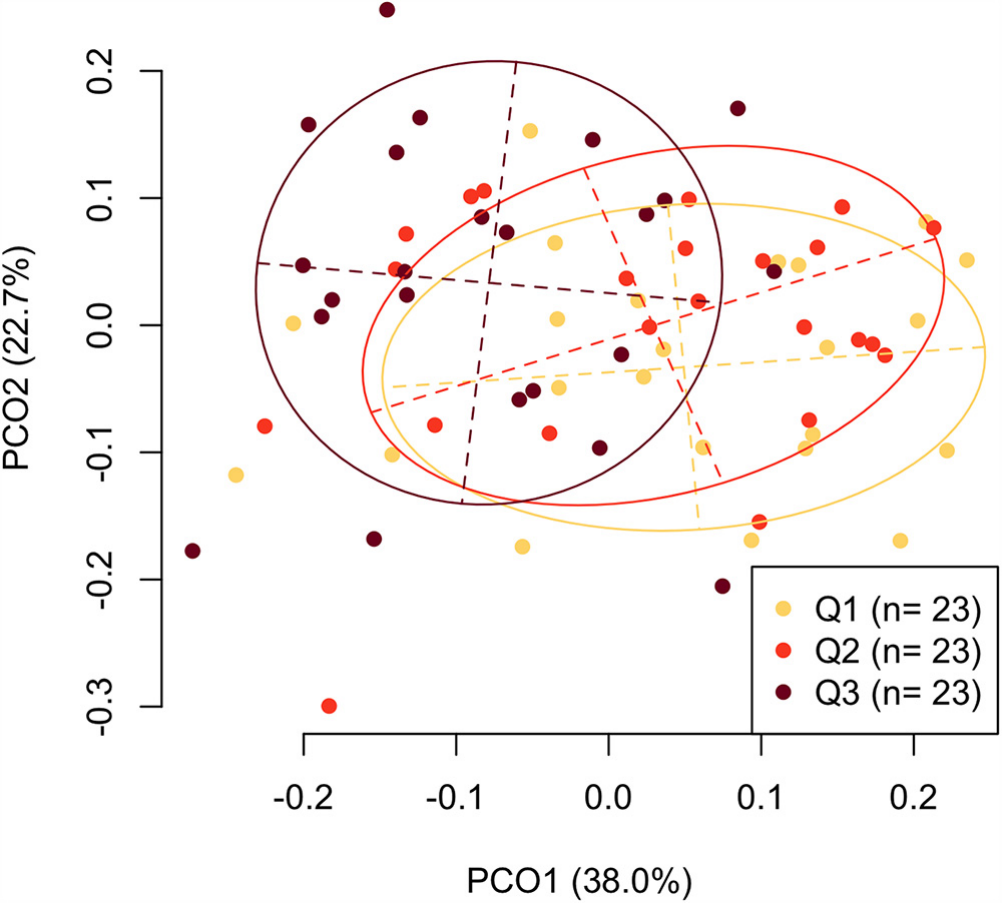
Principal-coordinate analysis plot showing the similarity relationship among the tongue microbiota compositions of 69 adult participants based on a weighted UniFrac distance metric. The samples collected from participants with three different levels of *n*-butyric acid concentration (classified by tertile; Q1 is lowest and Q3 is highest) in oral rinse samples are depicted as dots in different colors. The ellipses cover 67% of the samples belonging to each group. The axes explain 38.0% and 22.7% of the variance.

The bacterial composition of the tongue microbiota was compared among individuals whose oral *n*-butyric acid levels were classified based on their tertile values (Q1, ≤59.4 ng/mL; Q2, 59.4 to 151.1 ng/mL; Q3, >151.1 ng/mL). The general and dental conditions of individuals with three different concentrations of *n*-butyric acid are described in Table 1. A principal-coordinate analysis (PCoA) plot based on the weighted UniFrac metric demonstrated that the individuals exhibiting higher levels of *n*-butyric acid concentrations were localized in a relatively negative direction of principal coordinate 1 (Fig. 1). Permutational multivariate analysis of variance (PERMANOVA) for the variance of the weighted UniFrac distances confirmed that the bacterial composition was significantly different among individuals with the three levels of *n*-butyric acid concentrations (adjusted *P = *0.002), exhibiting the highest effect size (η^2^ = 0.100) among the six malodorous compounds assessed in this study (see Fig. S2 and Table S1 in the supplemental material). Of the other compounds, statistical differences according to the compound level were observed in propionic acid, phenol, and *p*-cresol in PERMANOVA (adjusted *P* = 0.005, 0.024, and 0.041, respectively) (Fig. S2).

**TABLE 1:** General and dental characteristics of participants with different *n*-butyric acid concentrations in oral rinse samples^*a*^

Characteristic	Value for those with *n*-butyric acid concn in tertile^*b*^			*P* ^*c*^
	Q1 (*n* = 23)	Q2 (*n* = 23)	Q3 (*n* = 23)	
Age	44.9 ± 9.0	45.4 ± 7.9	45.4 ± 8.4	0.80
No. of present teeth	29.0 ± 1.4	28.8 ± 1.3	28.5 ± 0.8	0.25
No. of DMF teeth	9.8 ± 5.1	11.7 ± 5.6	11.4 ± 6.2	0.39
Plaque index	0.76 ± 0.45	0.94 ± 0.47	0.97 ± 0.40	0.08
Gingival index	0.31 ± 0.27	0.47 ± 0.34	0.44 ± 0.26	0.09
Mean PPD (mm)	1.99 ± 0.37	1.97 ± 0.39	1.98 ± 0.25	0.95
Mean CAL (mm)	2.28 ± 0.65	2.20 ± 0.65	2.23 ± 0.62	0.73

a DMF, decayed, missing, and filled; PPD, periodontal pocket depth; CAL, clinical attachment level.

b Participants were classified into three groups based on tertiles of *n*-butyric acid concentrations in oral rinse samples.

c Jonckheere’s trend test.

10.1128/msphere.00490-22.2 FIG S2 Principal-coordinate analysis plot showing the similarity relationship among the tongue microbiota composition of 69 adult participants based on a weighted UniFrac distance metric. The points corresponding to the participants with three different levels of five malodorous compounds (propionic acid, phenol, *p*-cresol, indole, and skatole) concentration (classified by tertile; Q1 is lowest and Q3 is highest) in oral rinse samples are depicted using different colors. The ellipses cover 67% of the samples belonging to each group. An effect size (η^2^) and an adjusted *P* value in PERMANOVA are described in the top right of each diagram. The axes explain 38.0% and 22.7% of the variance. Download FIG S2, PDF file, 0.7 MB. Copyright © 2022 Chen et al. 2022 Chen et al. https://creativecommons.org/licenses/by/4.0/ This content is distributed under the terms of the Creative Commons Attribution 4.0 International license.

10.1128/msphere.00490-22.6 TABLE S1 Concentrations of six malodorous compounds in oral rinse samples of 69 participants. Download Table S1, DOCX file, 0.03 MB. Copyright © 2022 Chen et al. 2022 Chen et al. https://creativecommons.org/licenses/by/4.0/ This content is distributed under the terms of the Creative Commons Attribution 4.0 International license.

**FIG 2:**
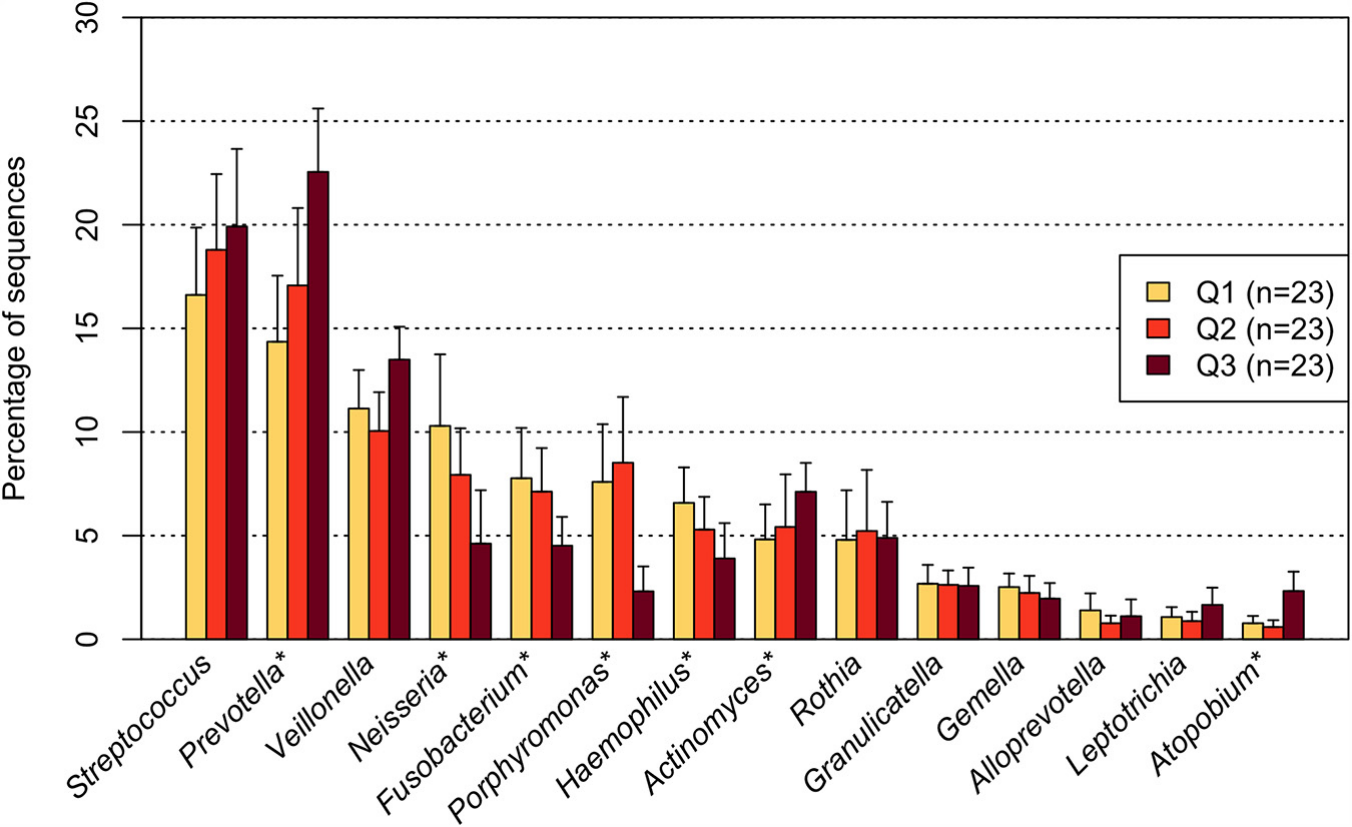
Relative abundances of predominant bacterial genera (mean relative abundance of >1%) of participants with different butyrate concentrations in their oral rinse samples. Significant differences were assessed using Jonckheere’s trend test. *, *P* < 0.05 after Benjamini-Hochberg adjustment for multiple comparisons.

The 14 predominant bacterial genera with mean relative abundances of >1% were commonly shared among most individuals regardless of their *n*-butyric acid levels (Fig. 2). In contrast, the relative abundances of *Prevotella*, *Actinomyces*, and *Atopobium* increased significantly with an increase in *n*-butyric acid concentrations, whereas *Neisseria*, *Fusobacterium*, *Porphyromonas*, and Haemophilus were significantly less predominant (adjusted *P < *0.05, Jonckheere’s trend test) (Fig. 2). At the species level, predominant bacterial taxa (mean relative abundance of >1%) such as Prevotella histicola, Veillonella atypica, and Streptococcus parasanguinis showed a significantly increasing trend in individuals with an increased concentration of *n*-butyric acid, whereas other predominant taxa, including Neisseria subflava and Porphyromonas pasteri, were present in significantly low proportions in the tongue microbiotas of individuals with an increased concentration of *n*-butyric acid (adjusted *P < *0.05, Jonckheere’s trend test) (Table 2).

**TABLE 2:** Relative abundances of bacterial species whose relative abundance showed significant increasing or decreasing trend according to *n*-butyric acid concentrations in the oral rinse samples^*a*^

Category and species (taxon ID)^*b*^	% relative abundance (mean ± SD) at *n*-butyrate concn tertile^*c*^		
	Q1 (*n* = 23)	Q2 (*n* = 23)	Q3 (*n* = 23)
Predominant at lower *n*-butyric acid concn			
Neisseria subflava (476)	10.27 ± 7.95	7.85 ± 5.18	4.6 ± 5.96
Porphyromonas pasteri (279)	6.71 ± 5.97	7.39 ± 6.5	1.68 ± 2.31
Streptococcus oralis subsp. *dentisani* (058)	0.78 ± 0.93	0.53 ± 0.74	0.07 ± 0.35
*Bergeyella* sp. (322)	0.12 ± 0.17	0.09 ± 0.12	0.01 ± 0.03
Predominant at higher *n*-butyric acid concn			
Streptococcus sp. (057)	0.07 ± 0.16	0.14 ± 0.24	0.31 ± 0.36
Prevotella salivae (307)	0.13 ± 0.25	0.32 ± 0.58	0.46 ± 0.48
Megasphaera micronuciformis (122)	0.26 ± 0.55	0.33 ± 0.46	0.99 ± 0.8
Streptococcus parasanguinis (411)	1.04 ± 1.58	0.88 ± 1.27	2.07 ± 1.88
Atopobium parvulum (723)	0.77 ± 0.81	0.59 ± 0.76	2.33 ± 2.16
Veillonella atypica (524)	1.85 ± 2.64	2.68 ± 2.56	4.02 ± 2.65
Prevotella histicola (298)	1.38 ± 3.41	2.35 ± 4.3	4.33 ± 5.68

a Significant differences among participants with three different *n*-butyrate concentrations were assessed for all taxa with a detection rate of >20% using Jonckheere’s trend test. Each *P* value was adjusted for multiple comparisons using the Benjamini-Hochberg method. Only bacterial taxa with an adjusted *P* value of <0.05 are shown.

b Oral taxon IDs in eHOMD are given in parentheses.

c Participants were classified into three groups based on tertiles of *n*-butyrate concentrations in oral rinse samples.

To assess whether butyric acid in the oral fluid alters the tongue microbiota composition, we cultivated the tongue microbiotas of 12 adults for 13 h in basal medium containing mucin (BMM), which allows a wide variety of oral indigenous taxa to grow, containing different concentrations of sodium butyrate (0, 0.8, 1.6, and 3.2 mM) (Fig. S3). The addition of *n-*butyric acid also drastically decreased the pH of the medium (pH 5.5 in the medium containing 3.2 mM *n-*butyric acid) and making it difficult to evaluate the effect of butyrate itself. Thus, sodium butyrate was used in this study, as it hardly affects the pH of the medium. We confirmed that all the 14 predominant genera could grow in the BMM, although a significant difference was observed in the relative abundance of *Gemella* before and after cultivation (adjusted *P < *0.01) (Fig. S4).

10.1128/msphere.00490-22.3 FIG S3 Total bacterial density of tongue microbiota samples before cultivation (precultivation) and those cultivated for 13 h with different concentrations of sodium butyrate (0, 0.8, 1.6, and 3.2 mM for total volume). Download FIG S3, PDF file, 0.2 MB. Copyright © 2022 Chen et al. 2022 Chen et al. https://creativecommons.org/licenses/by/4.0/ This content is distributed under the terms of the Creative Commons Attribution 4.0 International license.

10.1128/msphere.00490-22.4 FIG S4 Relative abundance of predominant bacterial genera before and after the 13-h cultivation of tongue microbiota. Significant differences were assessed using the Wilcoxon signed-rank test. **, *P* < 0.01 after the Benjamini-Hochberg adjustment for multiple comparisons. Download FIG S4, PDF file, 0.2 MB. Copyright © 2022 Chen et al. 2022 Chen et al. https://creativecommons.org/licenses/by/4.0/ This content is distributed under the terms of the Creative Commons Attribution 4.0 International license.

**FIG 3:**
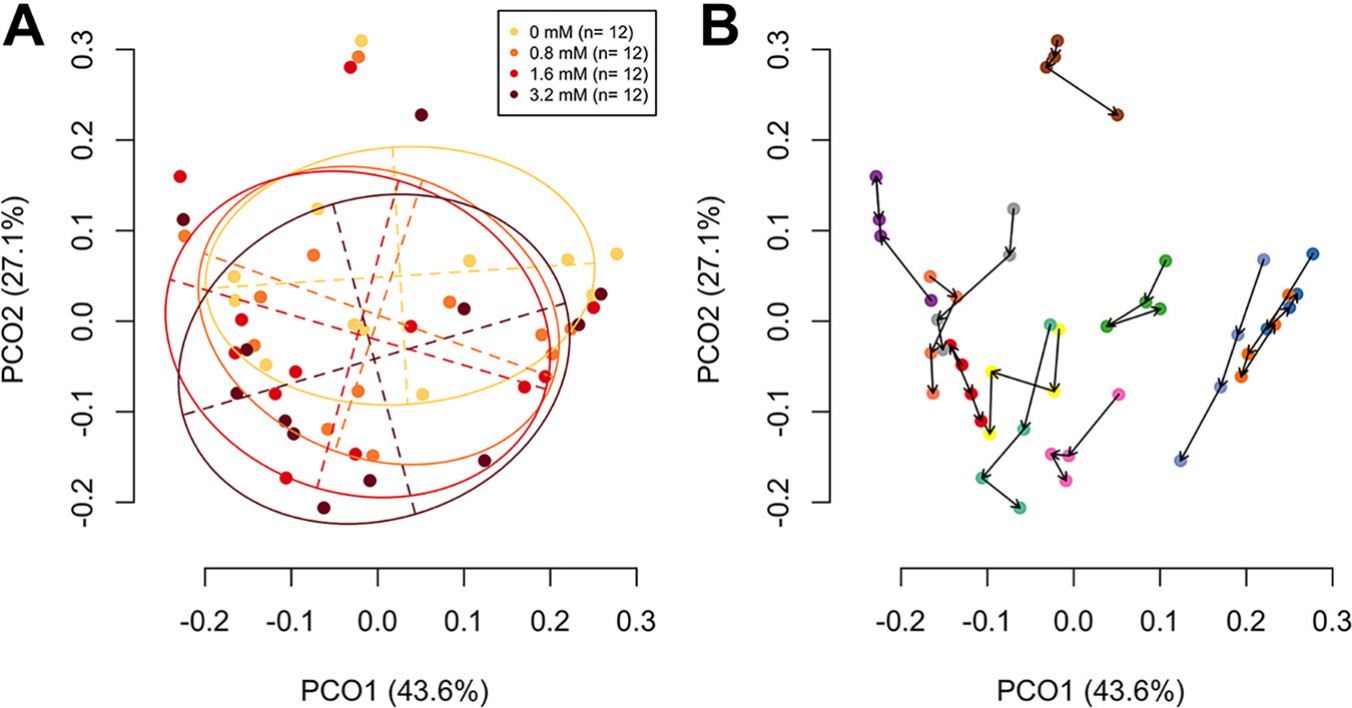
Principal-coordinate analysis plot indicating the similarity relationship among the tongue microbiota compositions of 12 adults after 13 h cultivation with four different levels of butyrate concentrations (0, 0.8, 1.6, and 3.2 mM for total volume) based on a weighted UniFrac distance metric. The axes explain 43.6% and 27.1% of the variance. (A) The samples to which different levels of butyrate concentration were added are depicted as dots in different colors. The ellipses cover 67% of the samples belonging to each group. (B) The samples collected from the same participants are depicted as dots in the same colors. The dots representing samples with addition of lower concentrations and those with higher concentrations (0, 0.8, 1.6, and 3.2 mM) are connected with black arrows.

No significant difference was observed in the total bacterial density among tongue microbiotas cultivated with different concentrations of sodium butyrate (Fig. S3). A PCoA plot based on the weighted UniFrac metric also indicated that the bacterial composition of the tongue microbiota cultivated with sodium butyrate was not distinct from that cultivated without sodium butyrate (Fig. 3A). However, when we focused on the microbiota shift in each individual, significant differences were observed in the data points for both principal coordinates 1 and 2 among the tongue microbiotas cultivated with different amounts of sodium butyrate (*P = *0.002 and *P < *0.001, respectively; Friedman test). The microbiota cultivated with an increased sodium butyrate concentration was located in a relatively negative direction of principal coordinates 1 and 2 in the plot, although several samples cultivated with the highest concentration of sodium butyrate (3.2 mM) were in a more positive direction of principal coordinate 1 than those cultivated with lower butyrate levels (Fig. 3B). PERMANOVA controlling permutations for interindividual variation also supported the observation that the addition of sodium butyrate significantly affected the bacterial composition of the tongue microbiota (*P* < 0.001).

**FIG 4:**
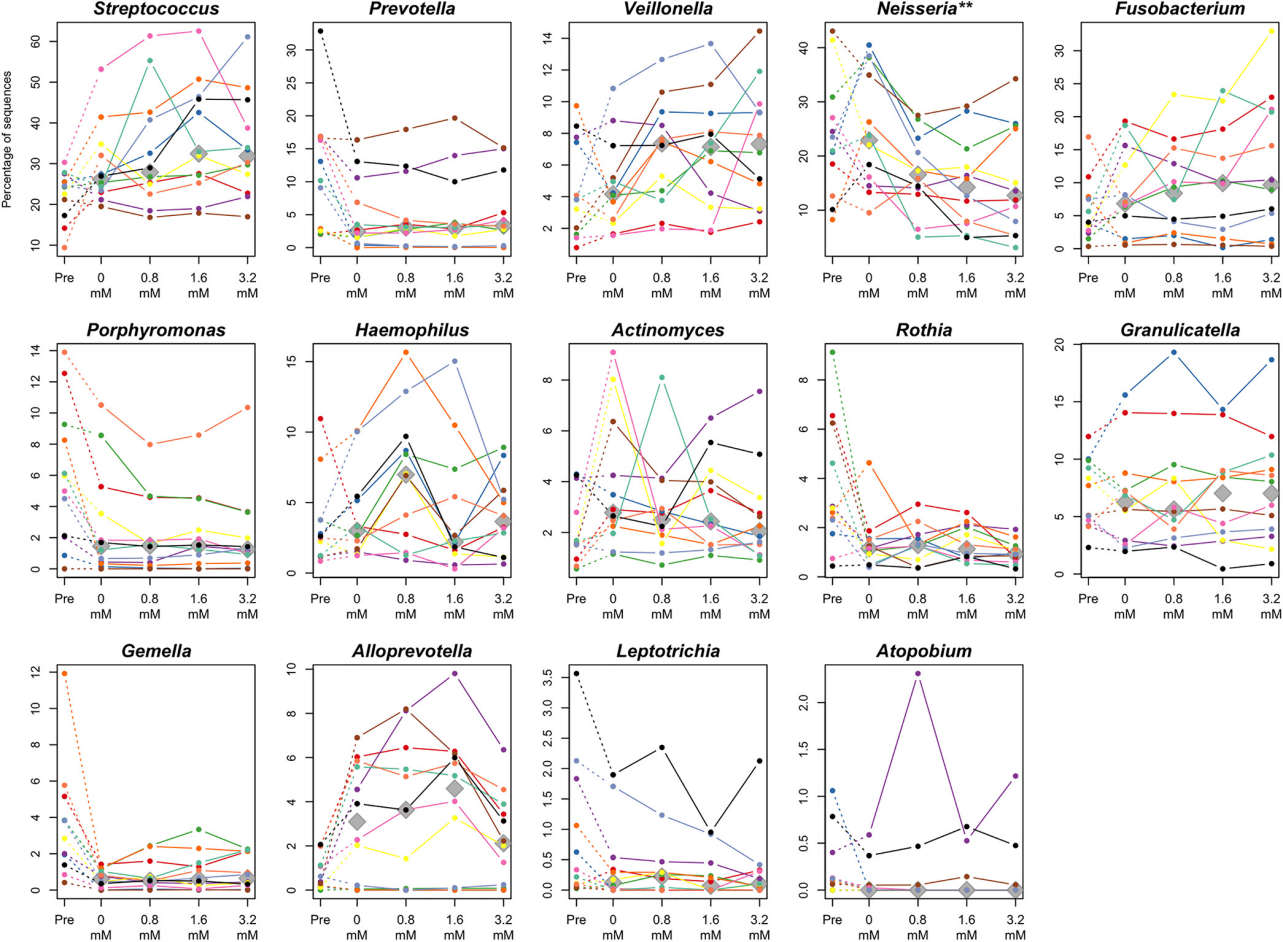
Relative abundance of predominant bacterial genera in the tongue microbiota of 12 participants (shown in different colors) before and after the 13-h cultivation with different sodium butyrate concentrations. Median relative abundances in each butyrate level are depicted with gray diamonds. Significant differences among the samples cultivated with different levels of sodium butyrate concentration were assessed using the Friedman test. **, *P* < 0.01, after the Benjamini-Hochberg adjustment for multiple comparisons.

**FIG 5:**
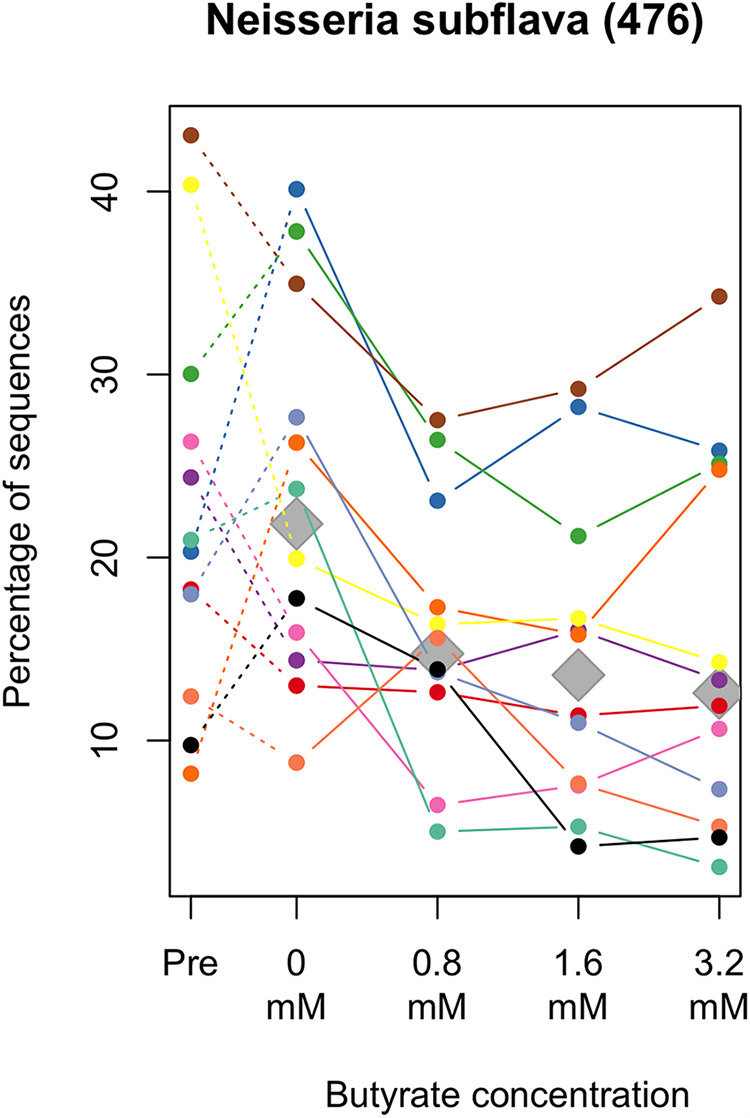
Relative abundance of *Neisseria subflava* in the tongue microbiota of 12 participants (shown in different colors) before and after the 13-h cultivation with different sodium butyrate concentrations. Of all taxa with detection rates of >20%, only the relative abundance of this species was significantly different after the 13-h cultivation according to varying sodium butyrate concentration. The number in parentheses following bacterial names indicates its oral taxon ID in eHOMD. Median relative abundances in each butyrate level are depicted with gray diamonds. Significant differences were assessed using the Friedman test (*P* < 0.05, after the Benjamini-Hochberg adjustment for multiple comparison).

Among the above-mentioned 14 predominant bacterial genera, *Neisseria* was significantly less predominant in the cultivated tongue microbiotas with high concentration of sodium butyrate (adjusted *P = *0.007, Friedman test) (Fig. 4), as observed in the tongue microbiotas of individuals with high *n*-butyric acid levels in the epidemiological study (Fig. 2). We subsequently explored bacterial species whose relative abundances were significantly different according to the added butyrate levels among all taxa, with a detection rate of >20%. A significant difference was observed in the relative abundance of *N. subflava* among the tongue microbiotas cultivated with different concentrations of sodium butyrate (adjusted *P = *0.028, Friedman test), and the median relative abundance was lower in samples cultivated with an increased butyrate concentration (Fig. 5).

## DISCUSSION

This study demonstrated that the tongue microbiotas of individuals with a high *n*-butyric acid level in their oral rinses contained higher relative abundances of *P. histicola*, *V. atypica*, and *S. parasanguinis* and lower relative abundances of *N. subflava* and *P. pasteri* (Table 2). A high ratio of predominant commensals, including *P. histicola*, *V. atypica*, and *S. parasanguinis*, to other predominant commensals, including *N. subflava* and *P. pasteri*, in the tongue microbiota has been implicated in poor dental conditions, such as more teeth with dental caries experience, poorer dental hygiene (7, 8), and fewer teeth (7), as well as increased risk of mortality from pneumonia in nursing home residents (15). Our other population-based study analyzing the saliva of 2,343 adults aged ≥40 years further indicated its association with an increase in age, body mass index (BMI), and current smoking (16). A salivary microbiota study of 268 healthy Dutch adults in combination with metabolome analysis demonstrated that an enrichment of *Prevotella* and *Veillonella* coincided with an increase in the abundance of dipeptides and high level of salivary albumin, implicating this community type as a potential dysbiotic pattern in early inflammatory states (17). An enrichment of *Prevotella* and *Veillonella* with a depletion of *Neisseria* in salivary bacterial populations was also observed in patients with inflammatory bowel disease compared with healthy controls (18). A recent study also indicated that a high relative abundance of *Prevotella* and *Veillonella* in the tongue microbiota was observed in patients with prolonged coronavirus disease 2019 (COVID-19) (19). These results suggest that the shifted equilibrium of common predominant commensals in individuals with an increased butyric acid concentration is a dysbiotic pattern of the tongue microbiota.

The cultivation of tongue microbiota samples in BMM further demonstrated that the addition of sodium butyrate significantly affected the bacterial composition of the tongue microbiota (Fig. 3). *N. subflava* was less predominant after cultivation with an increased addition of sodium butyrate (Fig. 5), which is consistent with the microbiota composition of individuals with a high level of *n*-butyric acid (Table 2). These results suggest the possibility that butyrate in the oral fluid contributes to the shifted equilibrium of the tongue microbiota. However, the differences in the cultivated microbiota attributable to the addition of sodium butyrate were not substantial (Fig. 3A) and were much smaller than the interindividual variations in the precultivated tongue microbiotas (Fig. 3B).

No statistically significant differences were observed between the other taxa, which are listed in Table 2. This inconsistency could be attributed to other environmental changes accompanied by an increased production of butyrate in the oral cavity. For example, butyric acid is an organic acid that can reduce pH in the oral environment. Most streptococci are acid-producing bacteria with acid tolerance, and a previous observational study indicated that low salivary pH is associated with a high proportion of Streptococcus species in salivary bacterial populations (17). Although sodium butyrate was added to the medium to maintain a neutral pH for the assessment of the effect of butyric acid itself, the addition of butyric acid, which results in a low pH, might reproduce the tongue microbiota composition in individuals with a high concentration of butyric acid. The concentration of propionic acid, which is also a short-chain fatty acid, was also high in oral cavities with high levels of *n-*butyric acid (Spearman correlation coefficient = 0.81, *P < *0.001) (Fig. S5). Further research that focuses on the ecological conditions or compounds shifting with butyric acid concentration in the oral cavity would be essential to identify other determinants involved in the equilibrium of predominant commensals of the tongue microbiota.

10.1128/msphere.00490-22.5 FIG S5 Relationship between the amounts of *n*-butyric acid and propionic acid in oral rinse samples of 69 adult participants. Download FIG S5, PDF file, 0.2 MB. Copyright © 2022 Chen et al. 2022 Chen et al. https://creativecommons.org/licenses/by/4.0/ This content is distributed under the terms of the Creative Commons Attribution 4.0 International license.

The intraoral concentration of butyrate has been reported to be higher in individuals with poor dental conditions, such as gingivitis and periodontitis (10, 20). Individuals with a high *n*-butyric acid level showed a high level of dental plaque and gingival indices, although these trends were not statistically significant in our small sample size (Table 1). Among the samples collected from various oral sites, dental plaque fluid (approximately 8 mM) (21) and gingival crevicular fluid (~5 mM) (10, 22) contain high concentrations of butyric acid. Considering that P. gingivalis and F. nucleatum, which are well-known oral butyrate producers, prefer to colonize gingival crevices among the various oral niches, it is reasonable to assume that butyric acid in the oral fluid is primarily derived from the tooth-associated microbial community. However, it is also likely that the microbial communities in other oral niches are involved in butyrate production. Although this study was unable to identify the bacterial taxa responsible for butyric acid production in the oral fluid, our results imply that a high butyric acid production in the oral cavity with poorer conditions would be associated with the bacterial composition of ingested tongue microbiota.

Antimicrobial effects of butyric acid have been reported for C. jejuni (11), H. pylori (12), and oral Streptococcus species, including S. gordonii and S. mutans (13). However, we did not observe growth inhibition of *N. subflava* ATCC 49275 in BMM with the addition of sodium butyrate (data not shown). Fatty acids are also involved in cross-feeding in the oral microbial communities (13, 23). The depletion of *N. subflava* in the tongue microbiota observed in this study might be caused by the alteration of the ecology owing to the addition of butyric acid rather than its bactericidal effect. Further studies are needed to elucidate the mechanism of the tongue microbiota shift caused by butyric acid in the oral cavity.

The cultivation of tongue microbiotas demonstrated a depletion of *N. subflava* with the addition of sodium butyrate to the medium, whereas several samples cultivated with the highest concentration (3.2 mM) of sodium butyrate contained a higher relative abundance of *N. subflava* than those cultivated with low butyrate concentrations (Fig. 5). A PCoA plot based on the weighted UniFrac metric also showed that several samples cultivated with the highest concentration of butyrate were not located farthest from those cultivated without butyrate, in the direction of principal coordinate 1 (Fig. 3B). These results suggest that the effect of butyrate on the tongue microbiota would not continue to increase linearly with the addition of butyrate.

Cultivation in BMM allowed the tongue microbiota to grow without an apparent shift in the bacterial community structure (Fig. S4). However, *Gemella* was significantly less predominant in the cultivated microbiota than in the microbiota prior to cultivation, suggesting that this cultivation condition was not preferable for this bacterium. It should be noted that the effect of butyric acid on the growth of *Gemella* species may not have been assessed accurately in this study due to its insufficient growth in the medium without the addition of butyric acid.

This study revealed that the shifted equilibrium of common predominant commensals in the tongue microbiota coincided with high butyric acid levels in the oral cavity. Our results also suggest the possibility that butyric acid affects the equilibrium of the tongue microbiota. These results add a new dimension to the ecology of the tongue microbiota. These findings would be helpful for determining the appropriate maintenance strategy for the tongue microbiota, in turn improving the bacterial composition of ingested microbial populations that reach distal organs, such as the lungs and gut.

## MATERIALS AND METHODS

### Ethics statement.

All participants understood the nature of the study and provided written informed consent. The Ethics Committee of Kyushu University approved the study design and procedure for obtaining informed consent for an observational epidemiological study for butyrate-associated microbiota (reference no. 27-363) and a cultivation study to assess the effect of sodium butyrate on oral microbiota composition (reference no. 21085-00).

### Observational epidemiological study.

Sixty-nine systemically healthy male adults aged 30 to 59 years were recruited from the employees of Kao Corporation and enrolled in an exploratory study of butyrate-associated microbiota. The participants refrained from consuming food or drink and mouth cleaning from the time they awoke until the time of sample collection, with the exception of drinking water, although that was also prohibited for 1 h before sample collection. The participants underwent dental examination, including assessment of dental hygiene status (plaque index), dental caries (number of decayed, missing, and filled teeth), gingivitis (gingival index), and periodontitis (mean periodontal pocket depth and clinical attachment level), followed by the collection of tongue microbiota and oral rinse samples. Tongue microbiota samples were collected using a sampling device based on a modified electric toothbrush (24). Briefly, a circular bonded-fiber fabric was attached to its round brush head, and the bristles were preliminarily removed. The head was placed and rotated at the center of the tongue dorsum, and the microbes that adhered to the fabric were collected. The fabric was peeled from the brush head and immersed in lysis buffer, from which microbiota DNA was extracted. The oral rinse samples were collected after gargling of 6 mL distilled water for 30 s. DNA was extracted from tongue microbiota samples using a bead-beating method, as described previously (7), and stored at −30°C until the bacterial community analysis, which is described below.

The concentrations of six malodorous compounds (*n*-butyric acid, propionic acid, phenol, *p*-cresol, indole, and skatole) in the oral rinse samples were estimated by gas chromatography-mass spectrometry (GC-MS) using an Agilent 6890/5973 GC/MSD system (Agilent Technologies, Palo Alto, CA). One milliliter of the oral rinse samples was mixed well with sodium chloride (0.5 g) and hexane/diethyl ether (2 mL, 1:1). After centrifugation, the supernatant was retrieved, centrifuged using a polytetrafluoroethylene filter, and used for chromatography analysis. Chromatographic separation was carried out using a DB-WAX column (30 m by 0.25 mm; inside diameter, 0.25 μm; Agilent Technologies). Helium was used as the carrier gas. The stepwise thermal conditions were as follows: the temperature was increased to and maintained at 40°C for 1 min; then, the temperature was increased to 70°C at a rate of 6°C/min. The temperature was then increased to 240°C at a rate of 3°C/min. The mass spectrometer was set to the selected ion-monitoring mode. The absolute abundance of each compound was determined using a standard curve prepared using a defined concentration of the analytes.

### Microbiota cultivation approach.

Fifteen systemically healthy adults (nine men and six women) aged 25 to 40 years were recruited from the students and staff of the Faculty of Dental Science, Kyushu University, excluding those who had consumed antibiotics within a month preceding sample collection or who had evident tooth decay or severe periodontitis. The participants refrained from consuming food or drinks, except for water, within 1 h before sample collection. Tongue microbiota samples from each participant were collected using a sampling device based on a modified electric toothbrush (7).

The collected tongue microbiota samples were inoculated into 2.5 mL BMM, which enables the cultivation of a wide variety of indigenous oral taxa (25, 26). BMM (pH 7.5) contains 2.5 g/L porcine gastric mucin (type III; Sigma Chemical, St. Louis, MO), 2.0 g/L proteose peptone (BBL, Becton, Dickinson, Sparks, MD), 1.0 g/L Trypticase peptone (Becton, Dickinson), 1.0 g/L yeast extract (Becton, Dickinson), 0.5 g/L potassium chloride (Wako, Osaka, Japan), 0.1 g/L cysteine hydrochloride (Wako), 0.001 g/L hemin (Sigma Chemical), and 0.0002 g/L menadione (Sigma Chemical). After dispensing 200 μL precultivated samples, the remaining inoculated tongue microbiota (250 μL each) was cultivated at 37°C in 5% CO_2_ for 13 h in the absence or presence of three different concentrations (0.8 mM, 1.6 mM, and 3.2 mM final concentrations) of sodium butyrate (Wako). The amount of added butyric acid was determined based on the concentrations in saliva of 20 dental patients (0 to 2.94 mM) and seven patients with chronic periodontitis (0.31 to 1.37 mM) reported in previous studies (20, 27). Following cultivation, 200 μL of the cell suspension was collected, and DNA was extracted from each sample using a bead-beating method. The DNA samples were stored at −30°C until quantitative PCR and 16S rRNA gene sequencing analyses.

Quantitative PCR analysis of the total bacterial count of all pre- and postcultivated samples was performed using the primers 806F (5′-TTA GAT ACC CYG GTA GTC C-3′) and 926R (5′-CCG TCA ATT YCT TTG AGT TT-3′) (28) using a QuantiFast SYBR green PCR kit (Qiagen, Hilden, Germany) according to the manufacturer’s instructions. The 16S rRNA gene of Porphyromonas pasteri was inserted into the vector plasmid pBluescript SKII(+) (Stratagene, La Jolla, CA) and used as a real-time control.

We inoculated the tongue microbiota attached to the fabric into cloudy BMM broth, which prevents accurate optical density OD measurement, and the inoculum was unable to be normalized for biomass in this study. However, the unnormalized inoculum could be a potential confounding factor for relative abundance at the endpoint. Therefore, we excluded the samples from three of the 15 individuals from the analysis to bring the bacterial amounts of all inocula within a range of 10-fold (10^5.55^ to 10^6.55^ copies of the 16S gene), based on the results of quantitative PCR analysis.

### Ion Torrent 16S rRNA gene sequencing analysis.

All 129 DNA samples (69 in the exploratory study and five from 12 individuals in the cultivation study) were subjected to 16S rRNA gene sequencing using a next-generation sequencer, Ion PGM (Thermo Fisher Scientific, Waltham, MA). The V1 and V2 regions of the 16S rRNA gene in each sample were amplified using the following primers: 8F (5′-AGA GTT TGA TYM TGG CTC AG-3′), with Ion Torrent adaptor A and the sample-specific 8-base tag sequence, and 338R (5′-TGC TGC CTC CCG TAG GAG T-3′) with the Ion Torrent trP1 adaptor sequence. PCR amplification, purification of each amplicon, pooling of amplicons, repurification, and quantification were performed as previously described (16). Emulsion PCR was performed using the Ion One Touch 2 system (Thermo Fisher Scientific), and sequencing was performed using an Ion PGM system (Thermo Fisher Scientific).

### Data processing.

Raw sequence reads were excluded from the analysis using R if they were ≤200 bases, if they were ≥700 bases, or if they did not include the correct forward and reverse primer sequences. The remaining reads were assigned to the appropriate sample by examining tag sequences using R, followed by trimming of tag and primer sequences and removal of reads with lengths of ≤240 bases. These quality-checked reads were further processed using the DADA2 pipeline version 1.21.0. (29), with default settings for Ion Torrent reads. The weighted UniFrac metric (30) was calculated to determine the dissimilarity between any pair of bacterial communities after rarefaction to 2,500 reads. We confirmed that the rarefaction curve for the number of unique sequences approached a plateau in each sample in this sequence depth (Fig. S1). The taxonomy of each denoised sequence was determined using BLAST against 998 oral bacterial 16S rRNA gene sequences in eHOMD (eHOMD 16S rRNA RefSeq version 15.1) (14). The nearest-neighbor taxon with ≥98.5% identity was selected as a candidate for each sequence. The taxonomy of the remaining undefined sequences was determined to the genus level using the RDP classifier with a minimum support threshold of 80%. The numbers of denoised sequences corresponding to the same taxa were combined, and the relative abundance of each taxon was calculated in R.

10.1128/msphere.00490-22.1 FIG S1 Rarefaction curve for a number of unique sequences per samples. A vertical line in the diagram indicates 2,500 reads. Download FIG S1, PDF file, 0.3 MB. Copyright © 2022 Chen et al. 2022 Chen et al. https://creativecommons.org/licenses/by/4.0/ This content is distributed under the terms of the Creative Commons Attribution 4.0 International license.

### Statistical analysis.

All statistical analyses were performed using R version 4.0.4 (31). Jonckheere’s trend test was conducted using the jonckheere.test function in the clinfun library (32) of R to evaluate increasing or decreasing trends in the general and dental conditions of participants and relative abundances of predominant bacterial genera and bacterial species, according to three butyrate concentration levels in the oral rinse samples (stratified by tertile). PERMANOVA based on the weighted UniFrac metric was used to assess the relationship between the three concentrations of the six malodorous compounds (stratified by tertile) and the tongue microbiota composition using the adonis function in the vegan library (33). The Wilcoxon signed-rank test was used to compare the relative abundances of the predominant genera before and after the cultivation of the tongue microbiota. The effect of sodium butyrate on the bacterial composition of the cultivated tongue microbiota was assessed via PERMANOVA based on the weighted UniFrac metric using the parameter “strata” to control permutations for interindividual differences. The Friedman test was conducted to compare the total bacterial density, data points for principal coordinates 1 and 2 in a principal-coordinate analysis plot, relative abundance of predominant bacterial genera, and bacterial species among the cultivated tongue microbiotas with four different concentrations of sodium butyrate. *P* values for multiple comparisons were adjusted using the Benjamini-Hochberg adjustment.

### Data availability.

The sequence data obtained in this study were deposited in the DDBJ Sequence Read Archive under accession no. DRA015084.

## References

[1] Schmidt TS, Hayward MR, Coelho LP, Li SS, Costea PI, Voigt AY, Wirbel J, Maistrenko OM, Alves RJ, Bergsten E, de Beaufort C, Sobhani I, Heintz-Buschart A, Sunagawa S, Zeller G, Wilmes P, Bork P. 2019. Extensive transmission of microbes along the gastrointestinal tract. Elife 8:e42693. doi:10.7554/eLife.42693. 30747106 PMC6424576

[2] Dickson RP, Erb-Downward JR, Freeman CM, McCloskey L, Falkowski NR, Huffnagle GB, Curtis JL. 2017. Bacterial topography of the healthy human lower respiratory tract. mBio 8:e02287-16. doi:10.1128/mBio.02287-16. 28196961 PMC5312084

[3] Huffnagle GB, Dickson RP, Lukacs NW. 2017. The respiratory tract microbiome and lung inflammation: a two-way street. Mucosal Immunol 10:299–306. doi:10.1038/mi.2016.108. 27966551 PMC5765541

[4] Zhou Y, Gao H, Mihindukulasuriya KA, La Rosa PS, Wylie KM, Vishnivetskaya T, Podar M, Warner B, Tarr PI, Nelson DE, Fortenberry JD, Holland MJ, Burr SE, Shannon WD, Sodergren E, Weinstock GM. 2013. Biogeography of the ecosystems of the healthy human body. Genome Biol 14:R1. doi:10.1186/gb-2013-14-1-r1. 23316946 PMC4054670

[5] Segata N, Haake SK, Mannon P, Lemon KP, Waldron L, Gevers D, Huttenhower C, Izard J. 2012. Composition of the adult digestive tract bacterial microbiome based on seven mouth surfaces, tonsils, throat and stool samples. Genome Biol 13:R42. doi:10.1186/gb-2012-13-6-r42. 22698087 PMC3446314

[6] Mager DL, Ximenez-Fyvie LA, Haffajee AD, Socransky SS. 2003. Distribution of selected bacterial species on intraoral surfaces. J Clin Periodontol 30:644–654. doi:10.1034/j.1600-051x.2003.00376.x. 12834503

[7] Asakawa M, Takeshita T, Furuta M, Kageyama S, Takeuchi K, Hata J, Ninomiya T, Yamashita Y. 2018. Tongue microbiota and oral health status in community-dwelling elderly adults. mSphere 3:e00332-18. doi:10.1128/mSphere.00332-18. 30111628 PMC6094060

[8] Zhang DX, Takeshita T, Furuta M, Kageyama S, Asakawa M, Nambu K, Yamashita Y. 2021. Tongue microbiota composition and dental caries experience in primary school children. mSphere 6:e01252-20. doi:10.1128/mSphere.01252-20. 33910998 PMC8092142

[9] Kurita-Ochiai T, Fukushima K, Ochiai K. 1995. Volatile fatty acids, metabolic by-products of periodontopathic bacteria, inhibit lymphocyte proliferation and cytokine production. J Dent Res 74:1367–1373. doi:10.1177/00220345950740070801. 7560387

[10] Niederman R, Buyle-Bodin Y, Lu BY, Robinson P, Naleway C. 1997. Short-chain carboxylic acid concentration in human gingival crevicular fluid. J Dent Res 76:575–579. doi:10.1177/00220345970760010801. 9042080

[11] Van Deun K, Haesebrouck F, Van Immerseel F, Ducatelle R, Pasmans F. 2008. Short-chain fatty acids and L-lactate as feed additives to control Campylobacter jejuni infections in broilers. Avian Pathol 37:379–383. doi:10.1080/03079450802216603. 18622853

[12] Yonezawa H, Osaki T, Hanawa T, Kurata S, Zaman C, Woo TDH, Takahashi M, Matsubara S, Kawakami H, Ochiai K, Kamiya S. 2012. Destructive effects of butyrate on the cell envelope of Helicobacter pylori. J Med Microbiol 61:582–589. doi:10.1099/jmm.0.039040-0. 22194341

[13] Huang CB, Alimova Y, Myers TM, Ebersole JL. 2011. Short- and medium-chain fatty acids exhibit antimicrobial activity for oral microorganisms. Arch Oral Biol 56:650–654. doi:10.1016/j.archoralbio.2011.01.011. 21333271 PMC3119748

[14] Escapa IF, Chen T, Huang Y, Gajare P, Dewhirst FE, Lemon KP. 2018. New insights into human nostril microbiome from the expanded Human Oral Microbiome Database (eHOMD): a resource for the microbiome of the human aerodigestive eract. mSystems 3:e00187-18. doi:10.1128/mSystems.00187-18. PMC6280432 30534599

[15] Kageyama S, Takeshita T, Furuta M, Tomioka M, Asakawa M, Suma S, Takeuchi K, Shibata Y, Iwasa Y, Yamashita Y. 2018. Relationships of variations in the tongue microbiota and pneumonia mortality in nursing home residents. J Gerontol A Biol Sci Med Sci 73:1097–1102. doi:10.1093/gerona/glx205. 29053769

[16] Takeshita T, Kageyama S, Furuta M, Tsuboi H, Takeuchi K, Shibata Y, Shimazaki Y, Akifusa S, Ninomiya T, Kiyohara Y, Yamashita Y. 2016. Bacterial diversity in saliva and oral health-related conditions: the Hisayama Study. Sci Rep 6:22164. doi:10.1038/srep22164. 26907866 PMC4764907

[17] Zaura E, Brandt BW, Prodan A, Teixeira de Mattos MJ, Imangaliyev S, Kool J, Buijs MJ, Jagers FL, Hennequin-Hoenderdos NL, Slot DE, Nicu EA, Lagerweij MD, Janus MM, Fernandez-Gutierrez MM, Levin E, Krom BP, Brand HS, Veerman EC, Kleerebezem M, Loos BG, van der Weijden GA, Crielaard W, Keijser BJ. 2017. On the ecosystemic network of saliva in healthy young adults. ISME J 11:1218–1231. doi:10.1038/ismej.2016.199. 28072421 PMC5475835

[18] Said HS, Suda W, Nakagome S, Chinen H, Oshima K, Kim S, Kimura R, Iraha A, Ishida H, Fujita J, Mano S, Morita H, Dohi T, Oota H, Hattori M. 2013. Dysbiosis of salivary microbiota in inflammatory bowel disease and its association with oral immunological biomarkers. DNA Res 21:15–25. doi:10.1093/dnares/dst037. 24013298 PMC3925391

[19] Haran JP, Bradley E, Zeamer AL, Cincotta L, Salive MC, Dutta P, Mutaawe S, Anya O, Meza-Segura M, Moormann AM, Ward DV, McCormick BA, Bucci V. 2021. Inflammation-type dysbiosis of the oral microbiome associates with the duration of COVID-19 symptoms and long COVID. JCI Insight 6:e152346. doi:10.1172/jci.insight.152346. 34403368 PMC8564890

[20] Koike R, Nodomi K, Watanabe N, Ogata Y, Takeichi O, Takei M, Kaneko T, Tonogi M, Kotani AI, Imai K. 2020. Butyric acid in saliva of chronic periodontitis patients induces transcription of the EBV lytic switch activator BZLF1: a pilot study. In Vivo 34:587–594. doi:10.21873/invivo.11811. 32111757 PMC7157893

[21] Margolis HC, Duckworth JH, Moreno EC. 1988. Composition and buffer capacity of pooled starved plaque fluid from caries-free and caries-susceptible individuals. J Dent Res 67:1476–1482. doi:10.1177/00220345880670120701. 3198845

[22] Qiqiang L, Huanxin M, Xuejun G. 2012. Longitudinal study of volatile fatty acids in the gingival crevicular fluid of patients with periodontitis before and after nonsurgical therapy. J Periodontal Res 47:740–749. doi:10.1111/j.1600-0765.2012.01489.x. 22594616

[23] Kolenbrander PE, Andersen RN, Blehert DS, Egland PG, Foster JS, Palmer RJ, Jr. 2002. Communication among oral bacteria. Microbiol Mol Biol Rev 66:486–505. doi:10.1128/MMBR.66.3.486-505.2002. 12209001 PMC120797

[24] Yano Y. 2011. Components of oral malodor caused by aging and its origin. Shikoku Dent Res 24(1):1–13. (In Japanese.)

[25] Bradshaw DJ, Marsh PD, Schilling KM, Cummins D. 1996. A modified chemostat system to study the ecology of oral biofilms. J Appl Bacteriol 80:124–130. doi:10.1111/j.1365-2672.1996.tb03199.x. 8642010

[26] Monoi N, Ohta H, Morishima S, Ochiai Y. 2004. Development of in vitro biofilm model: artificial food supplementation in chemostat-type system. J Oral Biosci 46:27–36. doi:10.1016/S1349-0079(04)80010-0.

[27] Silwood CJ, Lynch E, Claxson AW, Grootveld MC. 2002. 1H and (13)C NMR spectroscopic analysis of human saliva. J Dent Res 81:422–427. doi:10.1177/154405910208100613. 12097436

[28] Takeshita T, Nakano Y, Kumagai T, Yasui M, Kamio N, Shibata Y, Shiota S, Yamashita Y. 2009. The ecological proportion of indigenous bacterial populations in saliva is correlated with oral health status. ISME J 3:65–78. doi:10.1038/ismej.2008.91. 18830275

[29] Callahan BJ, McMurdie PJ, Rosen MJ, Han AW, Johnson AJ, Holmes SP. 2016. DADA2: high-resolution sample inference from Illumina amplicon data. Nat Methods 13:581–583. doi:10.1038/nmeth.3869. 27214047 PMC4927377

[30] Lozupone C, Knight R. 2005. UniFrac: a new phylogenetic method for comparing microbial communities. Appl Environ Microbiol 71:8228–8235. doi:10.1128/AEM.71.12.8228-8235.2005. 16332807 PMC1317376

[31] R Core Team. 2021. R: a language and environment for statistical computing. R Foundation for Statistical Computing, Vienna, Austria. https://www.R-project.org/.

[32] Seshan VE. 2018. clinfun: clinical trial design and data analysis functions. R package version 1.0.15. https://CRAN.R-project.org/package=clinfun.

[33] Oksanen J, Blanchet FG, Friendly M, Kindt R, Legendre P, McGlinn D, Minchin PR, O'Hara RB, Simpson GL, Solymos P, Stevens MHH, Szoecs E, Wagner H. 2020. vegan: Community Ecology Package. R package version 2.5–7. https://cran.r-project.org/web/packages/vegan/index.html.

