# Supplementary figures and images for: Butyrate as a Potential Driver of a Dysbiotic Shift of the Tongue Microbiota
Source: mSphere. 2022 Dec 12;8(1):e00490-22. doi: 10.1128/msphere.00490-22 (PMC9942584; doi:10.1128/msphere.00490-22)

### Propionic acid

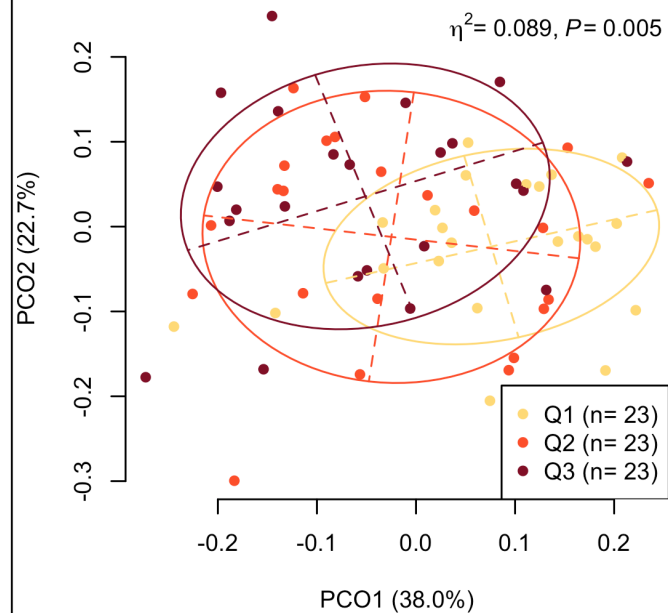

### Phenol

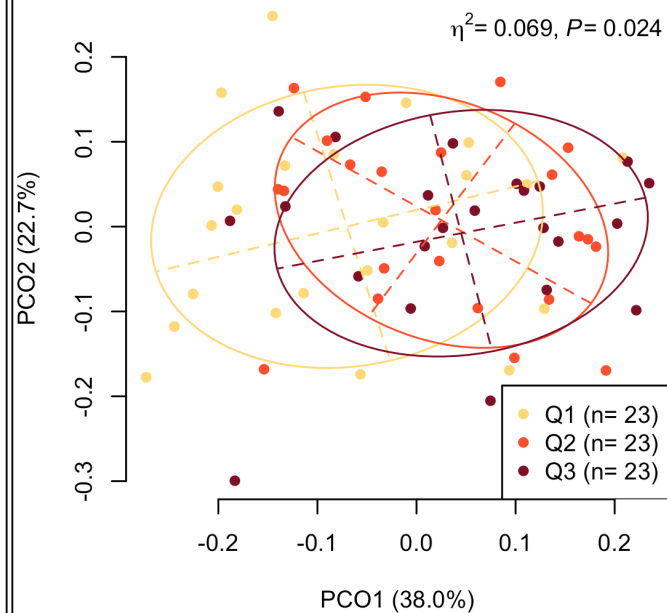

### p-Cresol

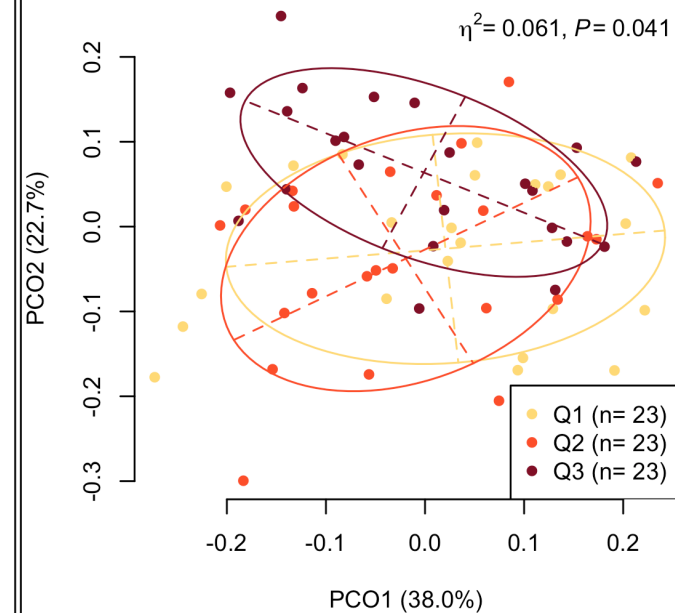

### Indole

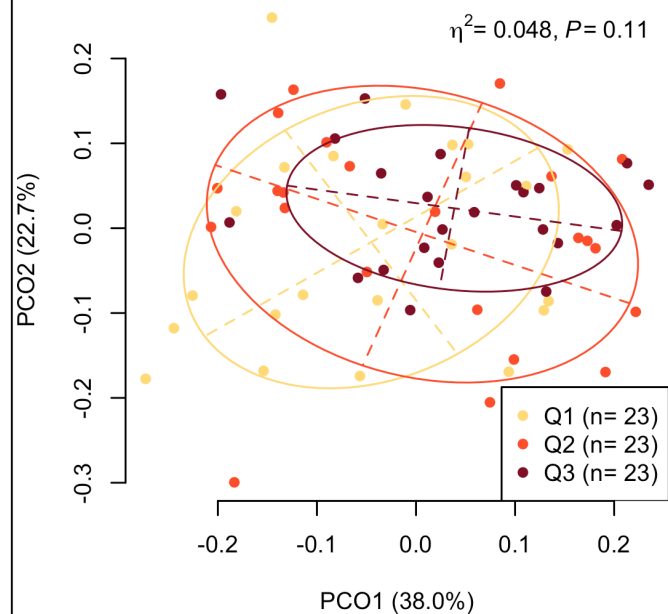

### Skatole

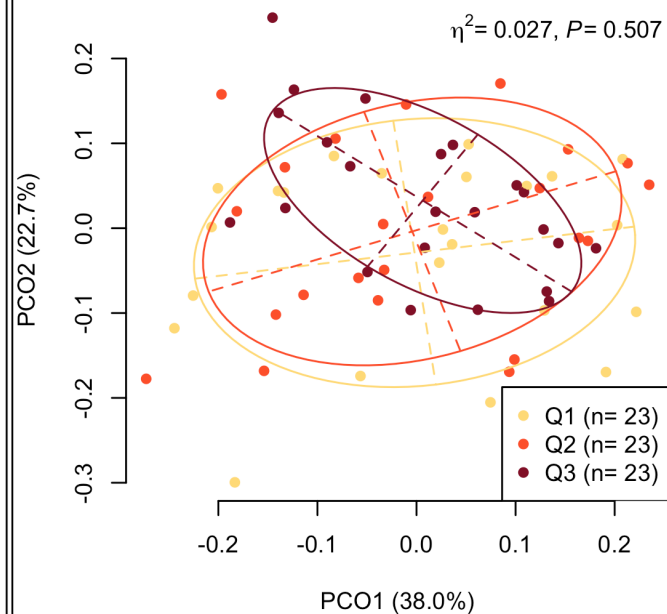

Fig. S2

Supplement: FIG S2 [file msphere.00490-22-s0002.pdf]

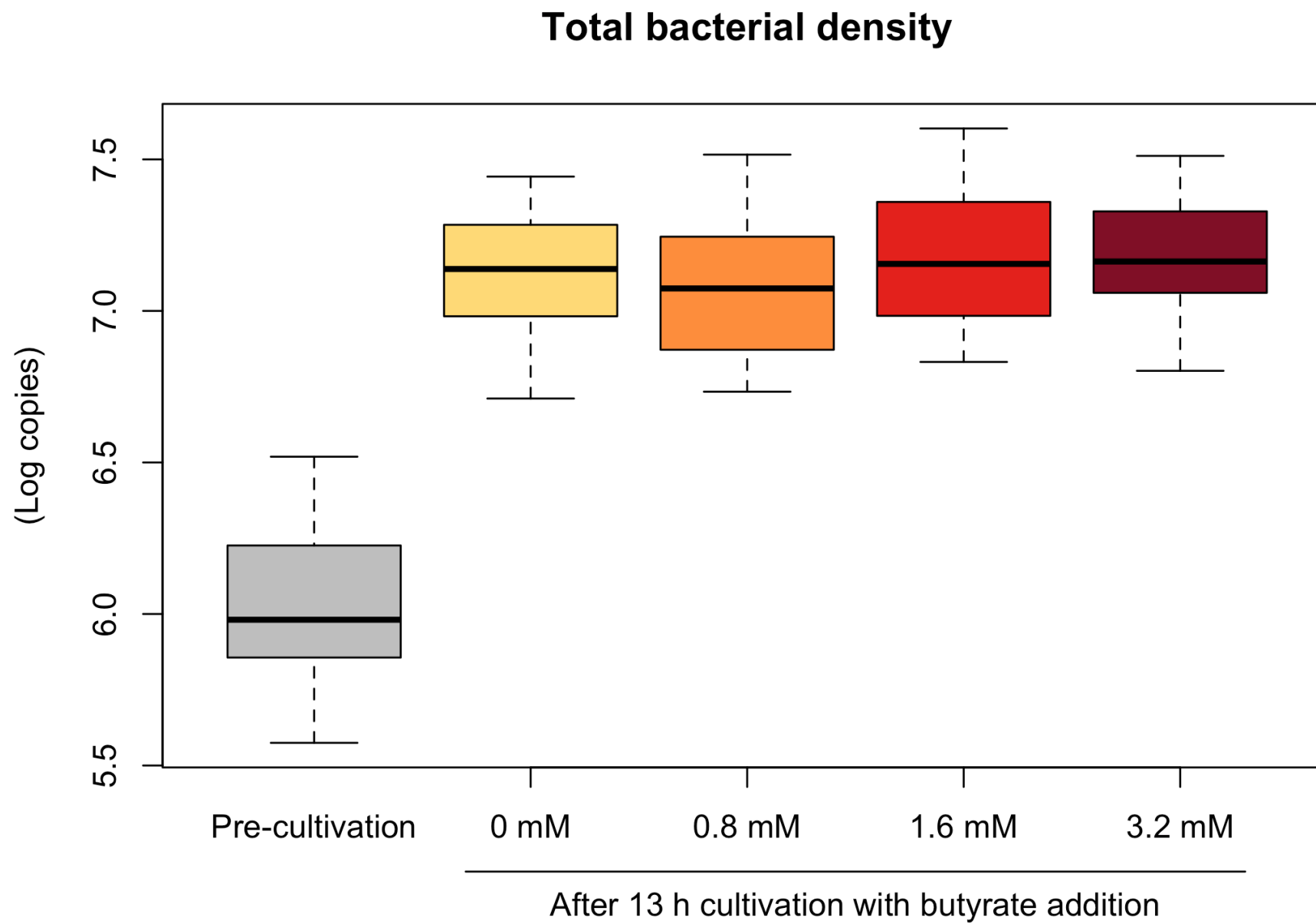

Fig. S3

Supplement: FIG S3 [file msphere.00490-22-s0003.pdf]

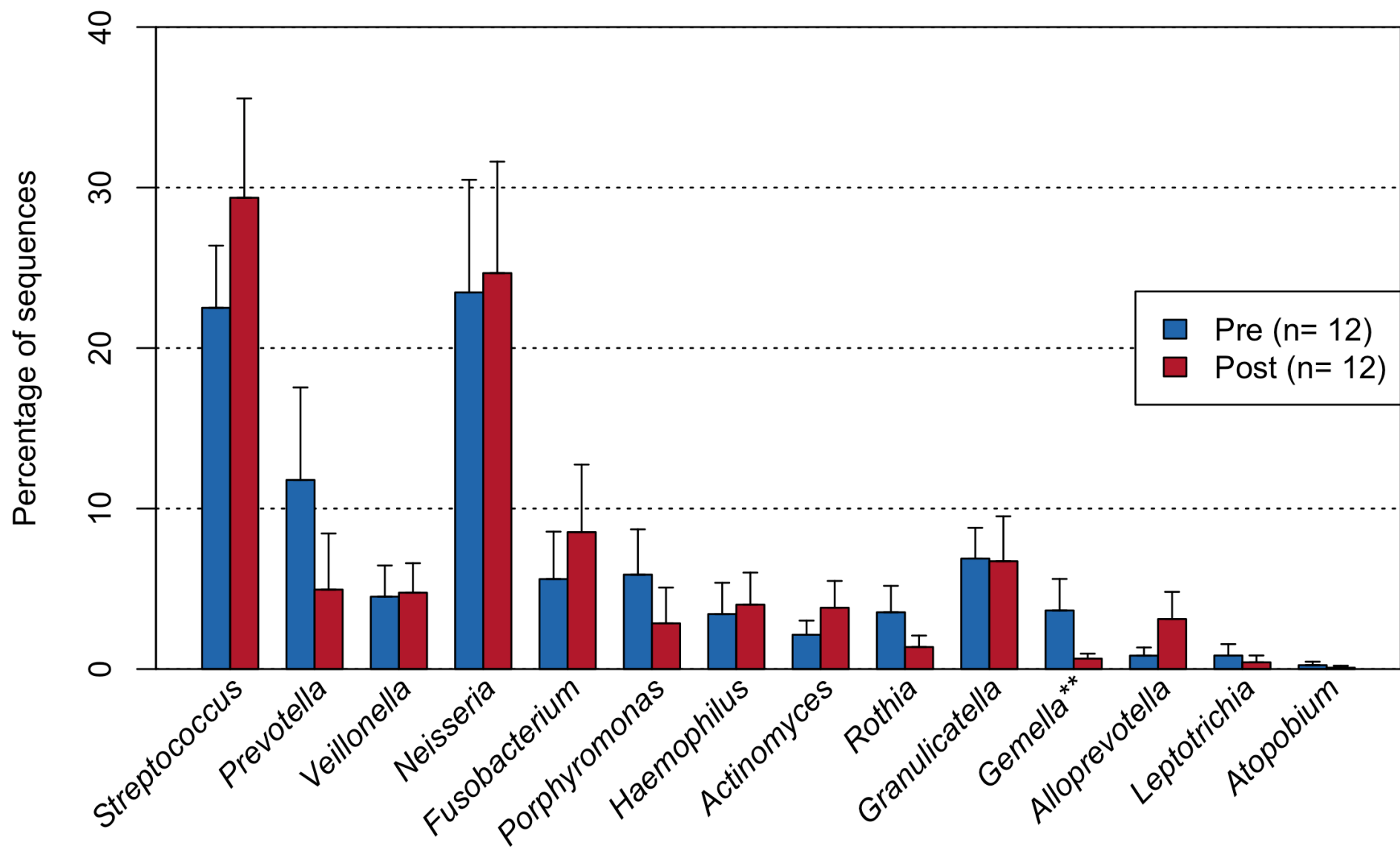

Fig. S4

Supplement: FIG S4 [file msphere.00490-22-s0004.pdf]

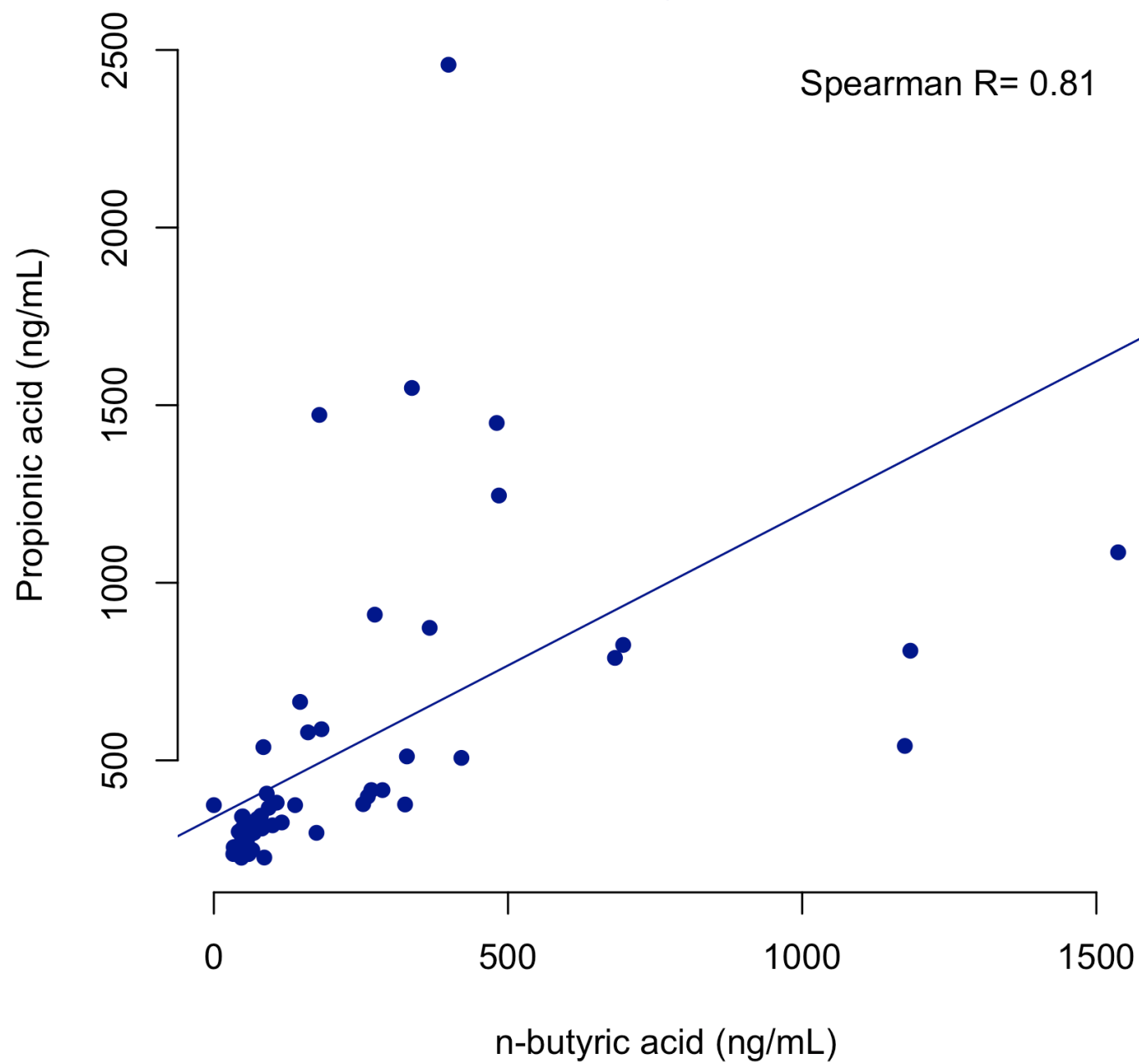

Fig. S5

Supplement: FIG S5 [file msphere.00490-22-s0005.pdf]

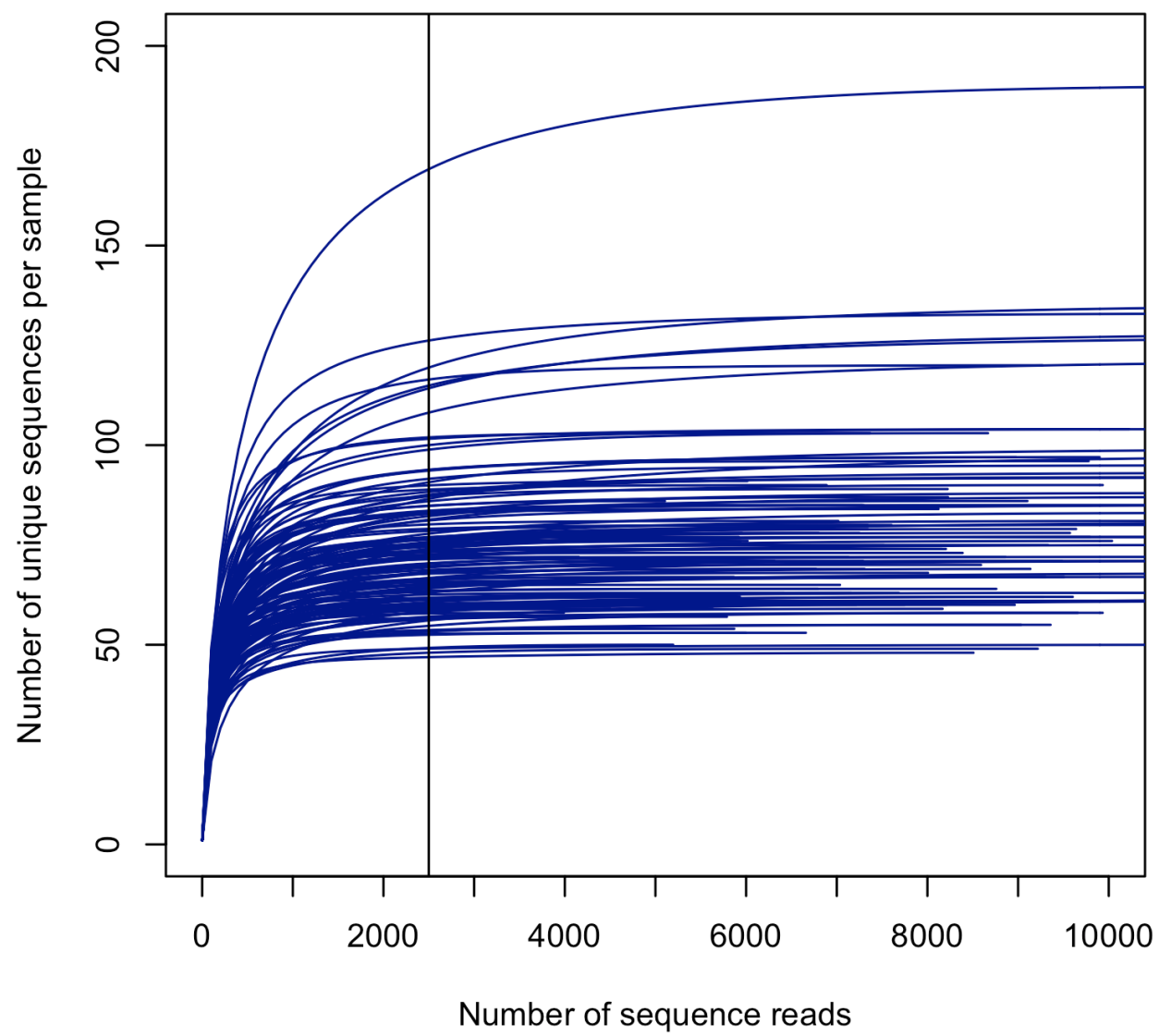

Fig. S1

Supplement: FIG S1 [file msphere.00490-22-s0001.pdf]
