# Supplementary material for: Butyrate as a Potential Driver of a Dysbiotic Shift of the Tongue Microbiota
Source: mSphere. 2022 Dec 12;8(1):e00490-22. doi: 10.1128/msphere.00490-22 (PMC9942584; doi:10.1128/msphere.00490-22)
Supplement: TABLE S1 [file msphere.00490-22-s0006.docx]

Supplemental table

Table S1 Concentrations of six malodorous compounds in oral rinse samples of 69 participants.

|  | Concentrations (ng/mL) | | | | |
| --- | --- | --- | --- | --- | --- |
|  | Minimum | 1st tertile | Median | 2nd tertile | Maximum |
| *n*-Butyric acid | 0 | 59.4 | 81.8 | 151.1 | 1537.1 |
| Propionic acid | 225.9 | 302.1 | 323.9 | 386.7 | 2663.8 |
| Phenol | 54.4 | 330.8 | 423.1 | 537.3 | 2199.2 |
| p-Cresol | 1.2 | 3.9 | 6.0 | 12.5 | 113.5 |
| Indole | 0 | 7.3 | 21.7 | 37.8 | 472.3 |
| Skatole | 0 | 0.5 | 0.8 | 1.2 | 32.1 |
